# Sequencing in Orthognathic Bimaxillary Surgery: Which Jaw Should Be Operated First? A Scoping Review

**DOI:** 10.3390/jcm12216826

**Published:** 2023-10-29

**Authors:** Lorenzo Trevisiol, Massimo Bersani, Guido Lobbia, Roberto Scirpo, Antonio D’Agostino

**Affiliations:** Department of Surgical Sciences, Dentistry, Gynaecology and Paediatrics, University of Verona, 37134 Verona, Italy; massimo.bersani@univr.it (M.B.); guido.lobbia@univr.it (G.L.); roberto.scirpo@univr.it (R.S.); antonio.dagostino@univr.it (A.D.)

**Keywords:** orthognathic surgery, bimaxillary surgery, surgical sequence in orthognathic surgery, mandible first, maxilla first, Le Fort I osteotomy, bilateral sagittal split osteotomy

## Abstract

Bimaxillary orthognathic surgery is widely used for the correction of dentoskeletal deformities. Surgery sequencing (maxilla or mandible first) remains debated, and guidelines and consensus are lacking. This scoping review summarizes the state of the art and compares the advantages and disadvantages of both approaches. The review was conducted following PRISMA-ScR guidelines. Three electronic databases (PubMed, Scopus, Web of Science) were searched using the PICO protocol and key words in orthognathic surgical sequencing. Four reviewers screened the records independently, and disagreement was resolved by consensus. A total of 23 records met the inclusion criteria. The advantages and disadvantages of the two approaches were compared and assessed for accuracy of reporting. Within the limitations of the present study, available evidence for the intrinsic advantages and the accuracy of the mandible-first sequence supports the choice of this approach in most cases. Nevertheless, each clinical case needs to be evaluated individually, as no dogmatic recommendations can be given for sequencing in bimaxillary orthognathic surgery.

## 1. Introduction

Since the introduction of internal rigid fixation, it has become the surgeon’s choice to decide which jaw to operate first in bimaxillary surgery for correcting malocclusion and improving facial appearance simultaneously [1,2]. In the past, at the time of wire fixation, the only way to perform bimaxillary surgery was to osteotomize the maxilla first because it was the only jaw that could be properly stabilized so as to be used as a reference point to safely reposition the mandible [1,3]. For this reason, most maxillofacial surgeons were trained to operate following a maxilla-first sequence.

With the introduction of titanium plates and screws, however, operating the mandible first has become a valid alternative, as it can be rigidly fixed and used as a stable reference point to reposition the maxilla. Surgeons can now easily stabilize both jaws effectively regardless of the sequence. Although the mandible-first sequence has attracted increasing interest among orthognathic surgeons, it is not known whether the mandible-first sequence is an alternative to the maxilla-first sequence out of convention or due to its real advantages.

To achieve optimal outcomes, surgical protocols should be closely followed to minimize the risk of complications and improve long-term results. Deciding which sequence to choose on a case-by-case basis in bimaxillary surgery is fundamental for improving the reliability of such a technically complex surgery and for achieving predictable outcomes [1,4,5].

Currently, very few studies summarize or compare the advantages and disadvantages of the two sequences. An extensive review of the topic is beyond the scope of the present article. The aim was to:

- Provide a summary of the available literature on sequencing in bimaxillary surgery;
- Compare the advantages and the disadvantages of both sequences;
- Provide clear indications for both sequences;
- Suggest a systematic study approach to those who intend to review the recent literature on this topic.

## 2. Materials and Methods

This scoping review was conducted according to PRISMA Extension for Scoping Reviews (PRISMA-Scr) guidelines [6]. This study was registered in the INPLASY platform. A summary of the protocol is provided in Table 1. A team of four reviewers differing in knowledge and experience in orthognathic surgery was recruited. Three electronic databases (PubMed, Scopus, Web of Science) were queried for publications on sequencing in bimaxillary orthognathic surgery published between 1977 and 2023. To do this, each reviewer drafted a list of keywords for the topic and created a search string following the PICO protocol (Table 2). The records were screened in a blind fashion by the four reviewers according to title and abstract. A calibration exercise was performed to ensure consensus among the reviewers. Studies with useful information about orthognathic sequencing were included, whereas studies on other concepts in orthognathics were excluded. After consensus on inclusion and exclusion criteria was reached, the full text of the records was analyzed, and a data charting form was created to identify which variables to extract. Each reviewer independently charted the data and discussed the results. By consensus, Table 3 was created to summarize the extrapolated data. A risk of bias assessment involving patients/measurements was performed using RevMan.

## 3. Results

**Figure 1:**
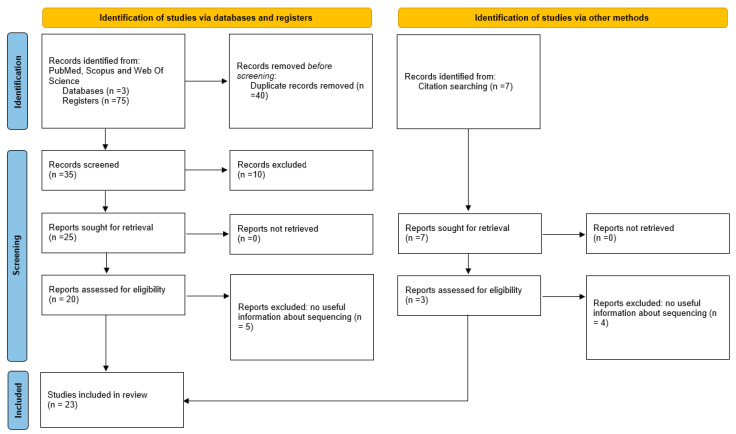
PRISMA flow diagram of inclusion/exclusion.

A total of 82 abstracts were retrieved from the three databases (75) and through in-text citations (7). After removal of duplicates, the remaining 42 abstracts were analyzed for relevance: consensus by all four reviewers was reached for 16; 6 were excluded, and 20 were listed as uncertain. The uncertain ones were further discussed: 7 were included in the study, and 13 were excluded. The final pool for this scoping review consisted of 23 records (Figure 1), which were fully read and summarized (Table 3).

A shift in study topics was noted over the years. While initially, there was interest in the advantages and the disadvantages of the mandible-first sequence with regard to surgical planning and surgical execution, the more recent studies investigated surgical accuracy and patient-specific implants (PSI) with both sequences. Most studies underscored the advantages of the mandible-first sequence, while few discussed the advantages of the maxilla-first sequence.

**Figure 2:**
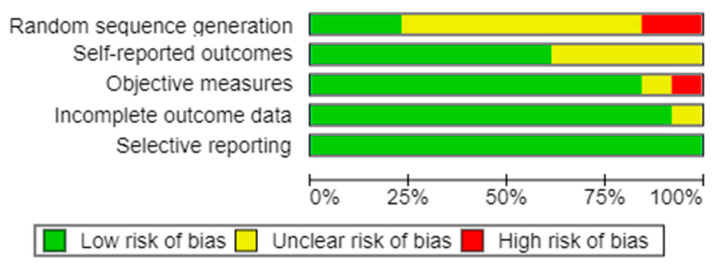
Risk of bias graph generated with RevMan.

**Figure 3:**
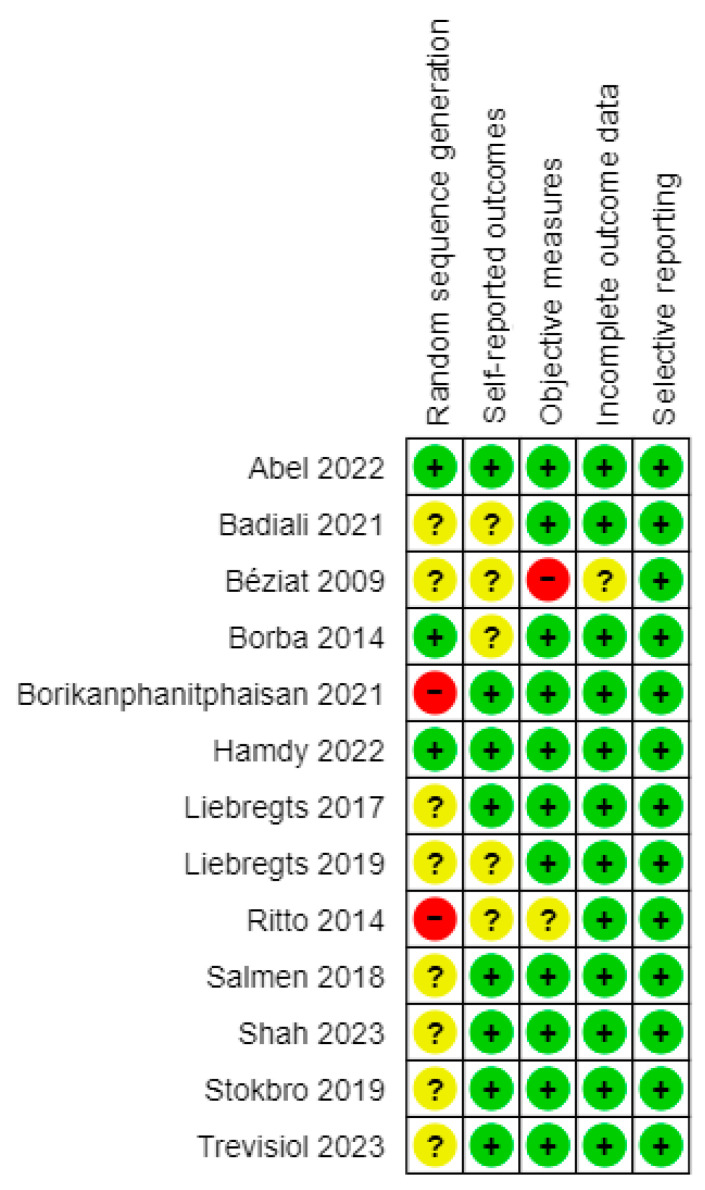
Risk of bias summary generated with RevMan. Evaluations are given according to RevMan risk of bias assessment criterias (“+” means low risk of bias; “?” means uncclear risk of bias; “–” means high risk of bias).

The risk of bias assessment showed an overall high quality of the studies, particularly for blinding and objectiveness of the measurements and completeness of data (Figure 2 and Figure 3). There were small issues with patient randomization, which is intrinsic to orthognathic surgery: treatment cannot be randomized, since it may affect the intervention outcome.

## 4. Discussion

Sequencing in bimaxillary orthognathic surgery has attracted intense debate, especially since the introduction of internal rigid fixation, which raised questions about the most appropriate sequence of osteotomies for correcting dentofacial deformities and achieving stable clinical results [2]. Interest was further kindled by Virtual Surgical Planning (VSP), which allows surgeons to plan sequences by their own preferences. Despite the longstanding debate over the advantages and the disadvantages of maxilla- and mandible-first sequences and despite the research conducted so far on the accuracy and surgical results of both approaches, consensus has yet to be reached, and neither the maxilla-first nor mandible-first sequence has been reported to be more advantageous.

This scoping review identified several key-points to consider before adopting the traditional or the inverted sequencing. What is beyond any doubt is that the decision about which jaw to operate on first must be taken at the time of surgical planning [2]. The entity of the defect to be treated (small/large sagittal movements), the direction of rotation of the maxillo-mandibular complex (clockwise or counter-clockwise), the importance of the reliability of the Centric Relation (CR) registration, and the need for maxillary segmentations and splint thickness are widely recognized as being decisive factors in the choice of surgical sequencing.

For example, the maxilla-first sequence is indicated in the clockwise rotation of the jaws, when a single-piece Le Fort I osteotomy is performed or when internal rigid fixation is unfeasible. This sequencing is also preferred in small maxillo-mandibular advancements or when maxillary impaction is required, which allows for the design of an intermediate splint that is thin enough to facilitate intraoperative rigid Maxillo-Mandibular Fixation (MMF) [14].

Choosing the mandible for the first osteotomy seems to be beneficial in the following scenarios. As stated by Posnick et. al., the mandible-first sequence is required when an accurate and reliable preoperative bite registration is not possible [10] and when concurrent Temporomandibular Joint (TMJ) surgery is performed before the orthognathic procedure, as it inevitably changes the position of the condyles [1,3]. Use of the non-operated maxilla as a fixed reference point allows for the accurate repositioning of the mandibular distal segment independently from the centric relation of the condyles during MMF, although the final position of the mandible (at MMF release) is dependent on the proper condyle repositioning in its fossa during plating [2,24].

Operating the mandible first is also advantageous in multi-segmental maxillary osteotomies in which maxillary fragments are oriented according to the mandibular arch; this provides an occlusal guide for achieving accurate and deep intercuspation, without the need for a palatal splint [1]. Perez et al. highlighted that a further advantage of the alternative sequence is that a large maxillo-mandibular advancement and counter-clockwise rotation can be easily performed: dental contacts are maximized, and the intermediate splint is thinner and fits better, resulting in better stability. The mandible-first sequence is to be preferred in maxillary down-grafting; otherwise, the intermediate splint would be too thick, and the TMJ might be displaced during surgery. A cleft lip also constitutes an indication for the mandible-first sequence [1,3,13,15].

Although the mandible-first sequence seems to be a reliable technique, it has several noticeable drawbacks. When a bad split of the mandible cannot be properly managed and the condyle cannot be passively placed in the fossae, the operation may need to be interrupted. Additionally, when a large maxillo-mandibular advancement with clockwise rotation is planned, the intermediate splint in a mandible-first sequence could create a large anterior open bite and make rigid MMF difficult to achieve; in such cases, a maxilla-first sequence is advised. Table 4 presents a summary of the factors to consider when selecting the sequence.

An additional consideration is the extent to which the choice of a surgical sequence could influence the treatment accuracy. Some authors stated that the two sequences are equally accurate, as both adhere to virtual surgical planning with comparable precision [5,19,21]. In contrast, other authors reported that the conventional sequence achieves more accurate results [12], with differences in specific surgical movements. Stokbro et al. found the mandible-first sequence to be more accurate when counter-clockwise (CCW) rotation was performed, while the conventional sequence apparently produced more accurate results when the rotation was clockwise (CW) [17].

Salmen et al. reported that the conventional sequence produced better esthetic outcomes due to the better accuracy of the vertical position of the upper incisors. In contrast, the mandible-first sequence was more accurate for the Pogonion vertical position [14]. Shah et al. and Abel et al. reported that the mandible-first sequence tended to underachieve maxillo-mandibular advancement in the sagittal direction [22,23]. It should be noted, however, that the discrepancies reported by all studies are clinically not significant, so both sequences may be considered equally accurate.

The development of Patient-Specific Implants (PSIs) with customized cutting guides and plates could represent a further improvement regarding this aspect. According to Badiali et al., PSIs produce clinically accurate results with both sequences [20]. Therefore, the choice of which jaw to operate first in a splintless bimaxillary surgery should follow the same principles as those stated in Table 4.

A final concern relates to the amount of mandibular autorotation required for the creation of the intermediate splint. It is postulated that the greater the amount of mouth opening, the greater the degree of inaccuracy. Recent studies, however, refute this postulate, as no correlation was found between mandibular autorotation and the inaccuracy of the final result [24].

In conclusion, based on the indications and the contraindications for sequencing, the mandible-first sequence appears to be the better choice in most bimaxillary surgery procedures. The surgeon’s personal experience and expertise play a central role in the choice of the surgical sequence, in which each case is evaluated individually, without dogmatic recommendations on which jaw should be operated first.

The present review has several limitations, including its vast and largely conceptual topic and the relatively small sample size. Prospective case-control studies are needed to establish the scientific soundness of the concepts underpinning orthognathic sequencing.

## Figures and Tables

**Table 1:** Summary of the study protocol according to the Prisma-ScR guidelines.

Protocol and registration	10.37766/inplasy2023.9.0022
Eligibility criteria	Peer-reviewed journal papers, publication period: January 1978–May 2023, Orthognathic sequence topic. Studies were excluded if they made no relevant mention or discussion of surgery sequence.
Information sources	PubMed, Scopus, Web of Science.
Search	PICO search strategy (Table 2).
Source of evidence and data charting	Four reviewers screened records in a blind fashion. Studies on orthognathic sequencing were included, whereas those on other concepts in orthognathic surgery were excluded. The reviewers independently charted the data and discussed the results to reach consensus.
Data items	Authors, year of publication, article format, study design, number and sex of patients (if available), and a summary of considerations/conclusions on orthognathic sequencing.
Synthesis of results	All full-text articles were retrieved and analyzed. Any additional reference that could contribute to the aim of the systematic review was included. The relevant information or concepts about surgical sequencing were extrapolated and synthesized.

**Table 2:** Keywords for the research string according to the PICO protocol.

Population	MeSH terms (1): Malocclusion OR Retrognathism OR Prognathisms OR Apertognathia OR Facial Asymmetries OR Micrognathisms Text word (2): Class II skeletal malocclusion OR Class III skeletal malocclusion OR Retrognathism OR Prognathism OR Apertognathia OR Facial asymmetry OR Open bite OR Maxillary excess OR Mandibular deficiency
Intervention	MeSH term (3): Orthognathic surgical procedure OR Lefort osteotomy OR Sagittal split ramus Osteotomy OR Maxillary osteotomy OR Mandibular osteotomy Text words (4): Orthognathic surgery OR Bilateral sagittal split osteotomy OR BSSO OR Intraoral vertical ramus osteotomy OR IVRO OR Le Fort I OR Bimaxillary surgery OR Setback OR Advancement OR Single jaw surgery OR Double jaw surgery OR Two jaw OR Two-jaw OR Double-jaw OR Single-jaw
Comparison	Text words (5): Mandible first OR Maxilla first OR Mandible-first OR Maxilla-first
Outcomes	Not applicable

Search algorithm: (1 OR 2 OR 3 OR 4) AND 5.

**Table 3:** Study Sample.

Authors	Year	Article Format	Study Design	Conclusions	Sample Size	Sequencing
**D. A. Cottrell and Wolford [7]**	1994	Original article	Conceptual	Mandible-first sequencing is advantageous in bimaxillary surgery when large mandibular advancements are required or maxillary walls are thin. The disadvantages are: mandatory rigid mandibular fixation with the condyle properly seated in the fossa; risk of surgery failure when a bad mandibular split occurs; risk of secondary posterior open bite when simultaneous advancement and CCW rotation are performed.	none	Mandible first
**J. Béziat [8]**	2009	Original article	Prospective cohort	Mandible-first sequencing is the preferable option in bimaxillary surgery, as it allows for the correction of potential errors of sagittal split osteotomy during Le Fort I positioning.	*n* = 50 (31 F, 19 M)	Mandible and maxilla first
**T. Turvey [4]**	2011	Communication to Editor	Conceptual	Sequencing in bimaxillary surgery is flexible and case-dependent. It should be based on accurate planning and preparation.	none	Maxilla first
**D. Perez et al. [1]**	2011	Original article	Conceptual	Performing mandibular osteotomies first is advantageous: 1. when down-grafting the posterior maxilla; 2. when unsure if bite registration is correct; 3. when intraoperative MMF in an interim position is difficult; 4. when fixation of the maxilla may not be rigid; 5. in concomitant TMJ surgery. Performing mandibular osteotomies first is disadvantageous due to the risk of unfavorable split.	none	Mandible first
**A. M. Borba et al. [9]**	2014	Original article	Prospective cohort	Mandible-first sequence is advised in patients with an unreliable centric occlusion (i.e., absence, loss or atrophy of the condyle).	*n* = 30 (21 F, 9 M)	Mandible first
**J. C. Posnick et al. [10]**	2014	Book chapter	Conceptual	Mandible-first approach is required when an accurate and reliable bite recording is not possible.	none	Mandible first
**F. G. Ritto [5]**	2014	Original article	Retrospective case-control	Performing maxilla-first or mandible-first approach in orthognathic sequence produces similar results with no significant differences in accuracy.	*n* = 40 (23 F, 17 M)	Mandible and maxilla first
**A. M. Borba et al. [2]**	2016	Original Article	Systematic review	Mandible-first sequencing is advantageous in certain conditions, such as unstable CR, counterclockwise rotations, and segmental maxillary surgery. Maxilla-first is to be preferred when clockwise rotations are planned. The mandible-first sequence seems to be more accurate, though additional supporting scientific data are needed.	none	Mandible first
**T. Iwai [11]**	2016	Short communication	Conceptual	Sequencing in bimaxillary orthognathic surgery is based on accurate pre-surgery planning; the maxilla-first approach is more widely used. In both cases, it is strongly recommended that one use straight locking mini-plates to achieve accurate condyle repositioning.	none	Mandible first
**D. Perez and E. Ellis [3]**	2016	Original article	Conceptual	The mandible-first approach is advantageous in the following situations: multi-piece maxillary osteotomy, large maxillo-mandibular advancement, counterclockwise rotation, unstable CR, concomitant joint surgery.	none	Mandible and maxilla first
**J. Liebregts et al. [12]**	2017	Original article	Retrospective cohort	In the majority of cases, the maxilla-first approach was more accurate in reproducing the 3D virtual planning.	*n* = 116 (80 F, 36 M)	Mandible and maxilla first
**D. Perez and A. Liddel [13]**	2017	Original article	Conceptual	The mandible-first sequence may be advantageous in cases such as large counterclockwise rotation, posterior maxilla down-grafting (i.e., class II and large open bite), large bimaxillary advancement, and when maxillary fixation may not be rigid (thin maxillary bone).	none	Mandible and maxilla first
**F. S. Salmen et al. [14]**	2017	Original article	Retrospective case-control	The maxilla-first sequence yields more accurate results in the upper incisor vertical position and better aesthetic outcomes, while mandible-first yields more accurate results in the Pogonion vertical position.	*n* = 32	Mandible and maxilla first
**S. Naran et al. [15]**	2018	Original article	Conceptual	The mandible-first sequence is suitable for: counterclockwise rotation of the occlusal plane, segmental maxillary osteotomies, cleft maxilla, down-grafting of the posterior maxilla, large maxillo-mandibular advancement, anterior open bite, inability to accurately register bite. The maxilla-first sequence is indicated in: clockwise rotation of the occlusal plane, single-piece Le Fort I osteotomy, impossible rigid mandible fixation, maxillary impaction, small maxillo-mandibular advancement.	none	Mandible and maxilla first
**J. Liebregts et al. [16]**	2019	Original article	Retrospective cohort	Maxilla- and mandible-first approaches produce comparable results in long-term skeletal stability. Whatever the sequencing, the mandible is always the less stable jaw.	*n* = 106 (73 F, 33 M)	Mandible and maxilla first
**K. Stokbro et al. [17]**	2019	Original article	Multicentric retrospective cohort	The maxilla-first approach seems to produce a more accurate maxillary repositioning. Counterclockwise rotation resulted in being more accurate when the mandible was operated first, while clockwise rotation showed better results in conventional sequencing.	*n* = 145 (98 F, 47 M)	Mandible and maxilla first
**E. V. Parente et al. [18]**	2019	Original article	Conceptual	Repositioning the mandible first in double jaw surgery produces better aesthetic results, as it allows for a more appropriate correction of facial asymmetry and a more accurate occlusal outcome. The surgical protocol can be applied regardless of an inaccurate preoperative CR registration.	none	Mandible first
**T. Borikanphanitphaisan et al. [19]**	2020	Original article	Retrospective cohort	In double jaw surgery, both mandible- and maxilla-first methods produce comparably accurate results, although the mandible-first procedure allows for more precise vertical dimension results.	*n* = 57 (37 F, 20 M)	Mandible and maxilla first
**G. Badiali et al. [20]**	2021	Original article	Prospective cohort	Repositioning the mandible first when using a patient-specific implants-guided protocol yields positive outcomes without sacrificing adjustability, thus reducing the risk of PSI inapplicability.	*n* = 22 (11 F, 11 M)	Mandible first
**M. Hamdy. Mahmoud and T.I. Elfaramawi [21]**	2022	Original article	Prospective case-control	The mandible-first surgical procedure is a reliable method to achieve a high maxillary stability in patients with class III malocclusion. The protocol produces results similar to the conventional approach.	*n* = 24 (11 F, 13 M)	Mandible and maxilla first
**A. R. Abel et al. [22]**	2022	Original article	Retrospective cohort	Mandible-first and maxilla-first surgical approaches seem to produce similar results in terms of accuracy in bimaxillary surgery with the use of PSIs, although an increased risk of posterior directional error may occur when repositioning the mandibula first.	*n* = 49 (24 F, 25 M)	Mandible and maxilla first
**B. Shah et al. [23]**	2023	Original article	Retrospective cohort	Both mandible-first and maxilla-first are reliable in accuracy. Although both tend to underachieve anterior-posterior advancement movements, maxilla-first seems to be the more accurate sequence.	*n* = 64 (N/A F, N/A M)	Mandible and maxilla first
**L. Trevisiol et al. [24]**	2023	Original article	Retrospective cohort	Mandible-first produces accurate results, even with movements considered unfavorable for this sequence. A slight underachievement of anterior-posterior advancement movements was noted.	*n* = 50 (37 F, 13 M)	Mandible first

**Table 4:** Factors to consider in surgical sequencing.

Key Factors	Mandible-First Sequence	Maxilla-First Sequence
Preoperative centric occlusion registration	Not relevant	Relevant
Le Fort I	Multisegment/One piece	One Piece
Maxillary vertical movement	Lengthening	Impaction
Occlusal plane manipulation	CCW Rotation	CW Rotation
Sagittal movement	Advancement	Small movements

CW—clockwise, CCW—counter-clockwise.

## Data Availability

No new data were created or analyzed in this study. Data sharing is not applicable.
